# Exploration of schizophrenia-related behavioral and molecular abnormalities in a mutant mouse model with a mutation in the TVV motif of the ErbB4 gene

**DOI:** 10.1186/s13041-025-01238-2

**Published:** 2025-10-09

**Authors:** Abdul Aziz Khan, Shuai Wang, Ziying Wang, Zainab Rehman, Lei Chen, Yifang Kuang, Xu Zhang, Yuting Li, Jiarun Yang, Jun Ye, Xianda Ma, Qian Zhao, Ying Ding, Tatsuo Suzuki, Zhaohui Lan, Weidong Li

**Affiliations:** 1 Bio-X Institutes, Key Laboratory for the Genetics of Development and Neuropsychiatric Disorders (Ministry of Education), Institute of Psychology and Behavioral Science, Center for Brain Health and Brain Technology, Global Institute of Future Technology, Shanghai Jiao Tong University, Shanghai, China; 2 Byrd Alzheimer’s Center and Research Institute, University of South Florida, Tampa, FL USA; 3 Shinshu University School of Medicine, Nagano, Japan; 4 WLA Laboratories, World Laureates Association, Shanghai, China

**Keywords:** Schizophrenia, ErbB4, Mutant-mouse, ErbB4-PSD-95-association, PSD-95, NMDAR2A hypofunction, GAD67

## Abstract

The ErbB4 gene is a schizophrenia (SCZ) risk gene that interacts with PSD-95 via its C-terminus, a connection disrupted in SCZ patients. To investigate the functional significance of this interaction, we generated a zygotic mutant mouse lacking the terminal valine “V” residue from the ErbB4 TVV motif. The homozygous (homo) mice exhibited disrupted ErbB4‒PSD-95 interactions and SCZ-relevant behavioral deficits, including impairments in motor function, sensory processing, and memory performance. Structural computational analysis further revealed that the mutation altered the structural conformation of the ErbB4 C-terminus, which affected its binding affinity for PSD-95. Mechanistically, the mutation led to up-regulated but less activation of ErbB4 and down-regulated but overactivation of PSD-95, possibly representing a failed compensatory response aiming to maintain the ErbB4-PSD-95 interaction. Additionally, homo mice presented NMDAR2A subunit specific hypofunction and reduced GAD67 expression. These findings highlight that the ErbB4–PSD–95 interaction is a critical molecular link in the synaptic dysfunction and behavioral abnormalities associated with SCZ.

**Supplementary Information:**

The online version contains supplementary material available at 10.1186/s13041-025-01238-2.

## Introduction

ErbB4 is expressed at the postsynaptic level in different regions (e.g., the hippocampus, amygdala, hypothalamus and cortex) in both the early embryonic and mature stages. It plays a major role in brain development, neurotransmission, and synaptic plasticity [1–3]. Accumulating evidence indicates that ErbB4 signaling contributes to the pathogenesis of several brain disorders [4–8], particularly schizophrenia (SCZ) [8, 9]. ErbB4 has consistently been associated with SCZ. Numerous human genetic studies and meta-analyses have consistently implicated ErbB4 in SCZ susceptibility [9, 10]. In addition to its genetic association, ERBB4 displays diverse pleiotropy across the endophenotypic traits associated with SCZ and is therefore considered an important gene for understanding the global disease dynamics of SCZ [11]. Genetic manipulation of ErbB4 in mice also induces psychiatric abnormalities [12–15]. At the molecular level, ErbB4 is a highly characterized receptor tyrosine kinase (RTK) that mediates neuregulin 1 (NRG1) ligand functions and interacts with key synaptic proteins [9]. The TVV motif of ErbB4 is particularly important for the ErbB4-PSD-95 interaction [16, 17]. Furthermore, ErbB4-PSD-95 interactions are specifically impaired in SCZ patients [18]. However, the role of the ErbB4‒PSD95 interaction in synaptic signaling and behavioral phenotypes has not been thoroughly explored.

ErbB4 is highly localized at excitatory synapses, where it contributes to the formation [2] and maturation [19] of glutamatergic synapses through its interaction with the PSD-95 protein at the postsynaptic density (PSD). ErbB4 plays a critical role in the development of GABAergic circuits by modulating synaptic transmission and enhancing GABA release in response to depolarization [1]. For example, the activation of ErbB4 by NRG1 leads to increased GABAergic transmission in the prefrontal cortex and basolateral amygdala (BLA), which is essential for excitatory and inhibitory transmission [1, 20, 21]. Both excitatory and inhibitory transmission control the excitatory/inhibitory (E/I) balance, and ErbB4 signaling sustains this E/I balance primarily by maintaining the integrity of the GABAergic system. Moreover, deletion of ErbB4 during adulthood does not affect inhibitory synapses onto excitatory neurons, nor does it alter excitatory synapses [1]. These previous findings pinpoint the diverse molecular functions of ErbB4 signaling and highlight its importance for normal neuronal transmission and cognitive function.

ErbB4 null mutant mice display social, sensory and memory-related behavioral deficits [12, 15]. In inducible knockout mice, tamoxifen treatments led to a reduced miniature inhibitory postsynaptic current (mIPSC) frequency and increased paired pulse ratios (PPRs), which indicates compromised GABA release efficacy [12]. Furthermore, regional-specific effects of Nrg1/ErbB4 signaling exist: Nrg1/ErbB4 signaling regulates inhibitory postsynaptic currents (IPSC) and enhances long-term potentiation (LTP) at Schaffer collateral-CA1 synapses [9], both of which are particularly critical for learning and memory [22]; in the basolateral amygdala (BLA), both ErbB4 and NRG-1 maintain GABAergic activity and anxiety-like behaviors [23]; and also sustain the plasticity of the parvalbumin (PV) neuronal network in the medial prefrontal cortex (mPFC), which is essential for fear extinction [24]. Collectively, these findings indicate that NRG1-ErbB4 signaling acts as a multimodal regulator of synaptic and circuit plasticity and that its disruption consequently contributes to behavioral deficits that are relevant to major psychiatric disorders.

The dual role of ErbB4 in modulating both excitatory (glutamatergic) and inhibitory (GABAergic) synaptic functions further emphasizes its importance in maintaining synaptic integrity and plasticity [25]. ErbB4 is unique because it has a C-terminal sequence (TVV) that conforms to the consensus site necessary for interacting with PDZ domains [16, 17]. The C-terminal tail region of ErbB4 directly interacts with PSD-95, facilitating the clustering of NMDA receptors at synapses and thereby enhancing excitatory synaptic transmission [26, 27]. However, there is no direct evidence demonstrating the functional role of the TVV motif in ErbB4, nor is it potentially impacting related behavioral phenotypes. Accordingly, we generated a novel mutant mouse by deleting the last valine “V” from the TVV motif of ErbB4 and explored the association of ErbB4 with PSD-95 and its role in synaptic signaling and SCZ-relevant behaviors.

## Methods

### Animals

The novel ErbB4 mutant mice were generated by Cyagen Company through zygotic modification as detailed in Additional file 1 and Additional file 2: Fig. S1A, B,further information is available from the author upon request..Heterozygous (Het) males and females were crossed to obtain all three genotypes: wild-type (Wild), het and homozygous (Homo). The mice were genotyped at 18‒21 days of weaning via the forward primer 5′-CGACTACTGGAACCACAGCCTGC-3′ and the reverse primer 5′-GCATCTCTACTGCTTCCACTGGAAGT-3′. Male adult mice (8–12 weeks) were used in the experiments. The mice were maintained under a 12 h light/dark cycle at the Laboratory Animal Center of Shanghai Jiao Tong University and had free access to food and water. All experiments were conducted following the guidelines of the institutional ethical committee (Ethical approval No. A2025211).

### Open field test

The mice were first acclimated to the experimental room for 1 h and then introduced carefully to explore the Plexiglas chamber, which measured 27.5 cm × 27.5 cm × 18 cm, for 20 min. Between trials, the chamber was cleaned with a 0.1% detergent solution and visually inspected for any leftover traces. The mouse movement data were automatically recorded via EthoVision XT software (V 10.0, Noldus, Wageningen, the Netherlands).

### Rotarod test

The mice were placed on a rotating rod cylinder (Ugo Basil, Italy) whose speed slowly accelerated from 4 to 40 r.p.m. over 5 min of testing. Each mouse underwent 3 trials with a 60-s rest interval between 2 consecutive trials, and the amount of time spent on the road was recorded. The time during which the mice remained on the rod was analyzed.

### Sociability test

In this test, the mice were first habituated to the experimental room for 1 h. To habituate the mice to the sociability test chamber box, they were individually placed in the central chamber with open doors and allowed to explore the whole chamber box (27.5 cm × 27.5 cm × 18 cm) for 10 min. In the test phase, a new C57BL/6J young female mouse was enclosed in a cylinder on one side of the chamber, while an empty cylinder was kept on the opposite side of the chamber. The experimental mice were individually allowed to explore the entire chamber box for 5 min. The time spent by experimental mice in the chamber containing female mice or the empty chamber was automatically recorded via EthoVision XT software (V 10.0, Noldus, Wageningen, the Netherlands).

### Elevated plus maze

To examine anxiety-like behavior, the mice were subjected to the elevated plus-maze test. The plus-shaped apparatus was elevated 74 cm above ground and consisted of two open arms (35 × 6 cm), two closed arms (35 × 6 × 22 cm), and a central zone (6 × 6 cm). Each mouse was individually placed at the center of the central zone facing an open arm to explore the maze for 10 min. Behavioral parameters, including the number of entries into the arms and time spent in each arm, were automatically recorded via EthoVision XT software (V 10.0, Noldus, Wageningen, the Netherlands). After each session, the apparatus was thoroughly sanitized with 75% ethanol.

### Tail suspension test

To assess depressive-like behaviors, the mice were subjected to the tail suspension test, wherein each mouse was gently suspended by the tail with adhesive tape so that the mice were positioned 50–60 cm above the surface to prevent them from reaching nearby objects. Behavioral activities were recorded for 6 min, typically under red light room lighting, and the immobility time and latency were first scored by an observer blinded to the groupings of the mice.

### Water maze test

We tested water maze activity via a plastic round container (120 cm diameter and 50 cm height) fitted in a dim light room with multiple visual cues on each sidewall. The tank was filled with 35 cm high water (20 ± 1.0 °C) that properly covered (approximately 0.5–1 cm) the round target platform (10 cm diameter). The swimming pattern variables (i.e., time in each quadrant, latency to reach the platform, and total distance) of the mice were recorded with a camera that was fitted on the top of the container and connected to a video tracking computer system.

All the experimental mice underwent 14 sessions of training over 7 consecutive days. In each of the four daily trials of two training sessions, the mice were placed in a random quadrant facing the wall and allowed to explore the hidden platform within 60 s. If they failed, they were guided toward the platform. In both cases, they were then allowed to rest on the platform for 30 s. Two consecutive trials had 30 s intervals. All the mice were first allowed to complete session one training, followed by session two. At days 3, 5, and 7, probe tests were conducted after the trials in which the mice were allowed to explore the hidden platform for 60 s. The total target quadrant time in the probe tests was used to assess performance. Mice with very slow swimming speeds (i.e., below 10 cm/s swimming speed) were considered floating and were not considered in the analysis.

### Prepulse inhibition (PPI)

The PPI test was performed via the SR-LAB™ Startle Response System (San Diego Instruments, USA). The mice were initially allowed to acclimate for 30 min to the laboratory environment, followed by another 5 min of acclimation to 65 dB background noise. Three types of testing sessions were then conducted in pseudorandom order with intertrial intervals of 10–20 s: (i) startle-alone trials (120 dB pulse, 40 ms), (ii) prepulse-pulse trials (20 ms prepulses of 76, 79, or 86 dB) that were presented 100 ms before the 120 dB startle pulse, and (iii) no-stimulus trials (background noise only). Startle amplitude (arbitrary units) and % PPI were calculated.

PPI (%) = [(Startle response without prepulse − Startle response with prepulse)/Startle response without prepulse] × 100.

### Forced swim test

In the forced swim test procedure, each mouse was gently placed in a 2-L beaker that contained water (25 ± 1 °C). The depth of the water was kept at 20 cm to ensure that the mice could not reach the bottom. Behaviors were recorded under red light conditions for 6 min. The immobility time, duration of floating or time in which the mice remained stationary, was then extracted from the videos by a researcher who was blinded to the experimental groups.

### Contextual fear conditioning test

The mice were exposed to a contextual fear conditioning paradigm chambers (Med Associates, Inc.). After 2 min of chamber exploration, each mouse received 3foot shocks of 0.75 mA intensity for 2 s with a 1 min interval between two sequential shocks. After shock training, the mice were returned to their cages for a 24-h rest, and the freezing behavior was observed for 5 min while the mice were reintroduced to the chambers (without shock) after 24 h and one week.

### Fear extinction test

The mice were first subjected to contextual fear conditioning trials on day 1, which consisted of three trials: S1, S2 and S3. Each trial is lost for 500 s while keeping a 3 h gap between each training trial session. In the training session, each mouse was kept in the chamber with a grid and received 0.7 mA foot shock for 2 s at 198 s, 278 s, 358 s, and 438 s intervals. After the trials, on days 2 and 3, the fear conditioning plus extinction group of mice was subjected to four extinction trials (E1-E4 on day 2 and E5-E8 on day 3). Each extinction trial lasts for 1800 s with a 2 h interval gap. In extinction trial sessions, the mice were kept in the same box previously used for fear conditioning on day 1 but received no foot shock. On day 4, the fear conditioning group and the fear conditioning plus extinction group were subjected to a retrieval test, which lasted for 500 s in the same box.

### Coimmunoprecipitation

Whole-brain lysates were prepared in RIPA buffer (Roche, Cat No. 4693132001) that contained 150 mM NaCl, 1.0% IGEPAL^®^ CA-630, 0.1% SDS, 0.5% sodium deoxycholate, and 50 mM Tris, pH 8.0. One tablet of protease inhibitor was dissolved in a total of 5 ml of buffer. The lysates were prepared within two minutes in three attempts using a PRO200 Homogenizer (Atkinson, USA) with an approximate 10 s gap to avoid denaturing protein interactions due to rotation heat. To remove cellular debris, the lysates were further centrifuged at 4 °C for 15 min at 12,000 r.p.m. speed using a Thermo Scientific™ Micro 17R centrifuge machine. The supernatants were collected in other precooled 1.5 ml Eppendorf tubes, and 2000 µg of protein was used in both the IP and IgG groups. The total volume was adjusted to 0.5 ml by adding the same homogenization buffer. The samples were then precleaned by adding 30 µl of precleaned A/G beads (Thermo Scientific Cat no. 88802) with gentle rotation at 4 °C for 4 h. The beads were washed twice with the washing buffer recommended by the manufacturer. Precleaned lysate was transferred to new precooled 1.5 ml Eppendorf tubes and immunoprecipitated for 14 h via an equal amount of primary antibodies: rabbit anti-ErbB4 (Cat No. 4795, CST), mouse anti-PSD-95 (Cat No. MAB1598, Sigma Aldrich), normal rabbit IgG (Cat No. 2729, CST), and mouse anti-IgG (Cat. No. A7028, Beyotime). After 2 h of initial incubation with primary antibodies, the lysate and antibody mixture were transferred into precooled new tubes containing 40 µl of precleaned magnetic beads. The mixture was gently rotated at 4 °C. The beads containing the associated attached proteins were collected and washed gently 3 times (5 min each) with slow vortexing while the samples were kept on ice. The attached protein complexes were eluted by adding 40 µl of 1X SDS buffer (dissolved in the same homogenization buffer) and keeping the samples in boiling water for 10 min. The samples were immunoblotted on a 10% gel (PG 112, Epizyme Biomedical Technology, Shanghai), and 45 µg of protein was used in the input control groups.

### Western blot analysis

Whole brains were homogenized in RIPA buffer containing 1 tablet of protease inhibitor and 1 tablet of phosphatase inhibitor (1 + 1 tablet/10 ml RIPA buffer). The lysates were subsequently centrifuged at 4 °C for 15 min at 12,000 r.p.m., after which total protein was quantified via a BCA kit (Cat No. 23227; Thermo Scientific). The proteins were separated via a 7.5% fast preparation WB gel (PG 111, Epizyme Biomedical Technology, Shanghai) and transferred onto a PVDF membrane. The membranes were blocked with quick blocking buffer (P0252 Beyotime) for 15 min and incubated at 4 °C overnight with the following primary antibodies: rabbit anti-ErbB4 (1:1000, Cat No. 4795, CST), mouse anti-PSD-95 (1:1000, Cat No. MAB1598, Sigma Aldrich), mouse anti-GAPDH (1:5000, Cat No. HC301, TransGen), rabbit anti-phospho-phospho-PSD-95 (1:1000, Cat. No. 45737, CST), rabbit anti-phospho-ErbB4 (1:1000, Cat No. 4757, CST), rabbit anti-NMDA receptor 2 A (1:1000, Cat. No. 4205, CST), rabbit anti-phospho-NMDA receptor 2 A (1:1000, Cat. No. 4206, CST), rabbit anti-GAD67 (1: 2000, Cat. No. ab239372, Abcam), rabbit anti-GAD67 (1: 1000, Cat. No. ab97739, Abcam), mouse anti-NMDAζ1 (1:300, Cat. No. sc-518053, Santa Cruz Biotechnology), and mouse VGLUT1 (1:500, Cat. No. NBP2-59329, Novus Bio). Following incubation with primary antibodies, the membranes were washed three times with 1X TBST for 10 min each time and incubated with DyLight 680 anti-mouse IgG (1:5000, Cat. No. 5470, CST) or DyLight 800 anti-rabbit IgG (1:5000, Cat. No. 5151, CST) secondary antibodies for 90 min at 37 °C. The membranes were rewashed three times before and after the addition of secondary antibodies, and the signals were visualized using enhanced chemiluminescence (ECL) (1:1 ratio, SB-WB012, ShareBio) for one minute with the Tanon-5200 Imaging System (Tanon Science & Technology).

### Cortical primary neuronal culturing

Primary neuronal cultures were prepared according to a previously established method [28] with slight modifications. Briefly, the cortices were isolated from newborn (P1) wild-type and homo mice and enzymatically dissociated at 37 °C for 20 min in dissociated solution (containing papain (10 U/mL, Solarbio) and DNase I (125 U/mL, Servicebio) dissolved in the culture medium). Dissociated cells were resuspended in culture medium and plated at a density of 1 × 106 per well in poly-D-lysine (PDL)-coated 6-well culture plates (Corning). After 4 h of incubation, the medium was replaced with prewarmed culture medium [neurobasal medium (Gibco) containing 2% B-27 (Gibco), 1% GlutaMAX (Gibco), and 1% penicillin/streptomycin (Gibco)]. Neurons were cultured at 37 °C in humidified 5% CO2, and half of the medium was replaced every 2–3 days. To inhibit the proliferation of glial cells, we also added AraC stock solution (1 mg/mL dissolved in Milli-Q water) to the wells at a concentration of 1 µg/mL for 12 h. Neurons from day 10 were further subjected to immunofluorescence staining experiments.

### Immunofluorescence staining

On day 10, primary neuronal cultures were first washed three times for 3–5 s with 1X PBS solution, fixed in 4% PFA for 15 min with gentle shaking, and then rewashed three times for 10 min each with 1X PBS. Cells were blocked with blocking solution (4% goat serum and 0.1% Triton X-100 dissolved in 1 × PBS) at RT for 1 h, followed by incubation with equal amounts (2.8 µg/200 µl solution) of primary anti-mouse ErbB4 and anti-rabbit PSD-95 antibodies at 4 °C overnight. After three sequential washes with IX-PBS, cells were incubated with fluorescently labeled secondary antibodies (1:500) for 90 min at 37 °C. Primary and secondary antibodies were diluted in P0103 (Beyotime) antibody solution. The samples were then washed three times with 1X PBS and stained with DAPI. Images were finally taken via a laser-scanning confocal microscope (SP8 Lightning, Leica) at 40X. Image analysis was performed via Fiji (ImageJ) software [29], and colocalization was quantified via the Coloc 2 plugin on the basis of Manders’ coefficients [30].

### Molecular docking

To investigate the molecular interaction and predict the binding affinity between ErbB4 and PSD-95, we docked the mouse ErbB4-C terminus with PSD-95 (domains 1 and 2) using the HADDOCK server [31]. The corresponding amino acid sequences were retrieved from UniProt [32] under accession numbers Q61527 (ErbB4) and E9Q9R9 (PSD-95). Model structures were prepared using Alphafold3 and i-TASSER [33, 34], incorporating domain-specific information based on previous findings [35, 36]. The top-ranked structural models were further selected for docking on the basis of multiple structural quality check parameters (Additional file 1: Table S1 and Additional file 1 and Additional file 3: Fig. S2A). Docked complexes were finally evaluated for predicted binding affinity and dissociation constant scores via the PROtein binDIng enerGY prediction (PRODIGY) server [37, 38].

### Molecular dynamics simulations

To explore the structural stability and flexibility of both wild-type ErbB4 + PSD-95 and mutant ErbB4 + PSD-95 protein complexes, we further performed Molecular Dynamics (MD) simulations on the best docked models (Additional file 1 and Additional file 3: Fig. S2B-E). These simulations help us to understand how the protein complexes behave over time under a nearly mimicked cellular environment and to examine how the mutation affects the protein structural stability and flexibility. The MD simulations were conducted using the Amber20 version software [39], using the FF14SB force field, which is a standard model that gives information about protein movement and interaction at the atomic level. The simulation systems were initially solvated in a TIP3P water box, mimicking a physiological water environment and neutralized with counterions [40]. To remove any unrealistic atomic clashes from models, we performed energy minimization steps (10000 cycles of the steepest descent algorithm [41] and 5000 cycles of the conjugate gradient algorithm [42]). The systems were then slowly heated to 300 K at a constant pressure of 1 atm first with weak restraint and then without restraint, and the production step was run for 100 ns with PMEMD.CUDA. The long-range electrostatic interaction was treated with the particle mesh Ewald algorithm [39] which is important for capturing realistic protein behavior. The MD trajectories were analyzed via the Amber20CPPTRAJ package [43]. All MD simulations were performed in triplicate. Three-dimensional protein models were made in PyMOL (2.5.5, Schrödinger, LLC), and multiple structural related post simulation analyses (root mean square deviation (RMSD), root mean square fluctuation (RMSF), hydrogen bond number, and radius of gyration (Rg) were made in Origin ProLAB v2021 from average values.

### Statistical analysis

Graphs were generated via GraphPad Prism version 8 software. All the data are presented as the means ± SEMs. Statistical analysis was performed via SigmaPlot version 14.0 software. Student’s t-test or Mann-Whitney t-test were used to compare two groups, while one-way ANOVA or ANOVA on ranks with Student-Newman-Keuls method as post hoc test were used for comparisons of multiple groups. A p-value of < 0.05 was considered statistically significant. The data are presented as the means ± SE.

## Results

### Behavioral deficits in ErbB4 mutant mice

**Fig. 1:**
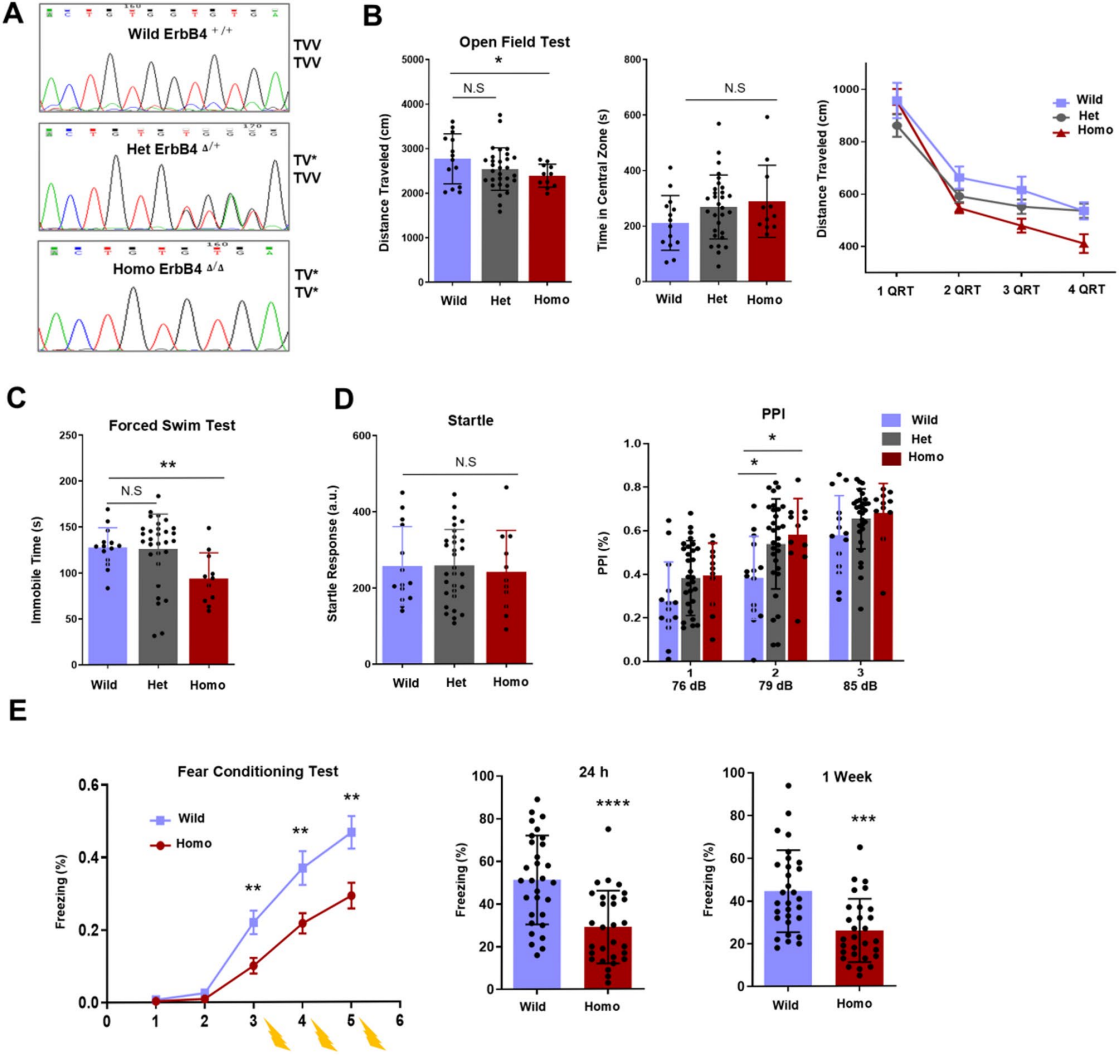
Behavioral abnormalities in ErbB4 mutant mice. Genotyping results show the GTG base pair which encodes last Valine (V) in the TVV motif region is missing in homo mutant mice (**A**). A decrease of total distance (**B**) was observed in homo mice in open field test and no significance was found in central zone (*p* < 0.05 and *p* > 0.05 respectively, ANOVA on ranks with Student-Newman-Keuls post hoc test). The immobility time of homo mice was decreased but no significance was observed in het mice (**C)**, (*p* < 0.01 and *p* > 0.05 respectively, ANOVA on ranks with Student-Newman-Keuls post hoc test). In **B** and **C** behavioral tests, same cohort of mice were used (Wild: *N* = 14, het: *N* = 30, and homo: *N* = 11). The startle response of all group’s mice was normal (**D**) but homo and het mice showed high average prepulse inhibition this suggests mutant mice has enhanced sensorimotor gating or high-level tolerance. Homo mice also exhibited spatial memory defects as shown by low freezing behavior in both 24 h and 1 week (**E**) fear conditioning tests (Wild: *N* = 30 and homo: *N* = 29, Student’s t-test, *p* < 0.0001 and *p* < 0.001 respectively). N = number of mice, * = *p* < 0.05, ** = *p* < 0.01, *** = *p* < 0.001, **** = *p* < 0.0001

To evaluate the functional consequences of ErbB4-PSD-95 dissociation in mutant mice, we first confirmed the model through genotyping and then performed multiple relevant behavioral analyses. The genotyping results revealed the deletion of the last valine “V” in the TVV motif of ErbB4 in the homo mutant mice (Fig. 1A). The mutation had no effect on body weight (Additional file 1 and Additional file 2: Fig. S1C). Compared with wild-type, homo mice were less active and exhibited motor impairments in the open field test (Fig. 1B), which suggests cortical dysfunctions. Homo mice were further less immobile than wild-type in the forced swim test (Fig. 1C). The mutation also caused hypersensitivity in the mice, as the homo mice presented a high percentage of prepulse inhibition at 79 dB (Fig. 1D). Since the hippocampus-dependent memory defects are common in SCZ patients. We also detected spatial memory defects in mutant mice, as shown by low freezing behavior in both the 24 h and 1week fear conditioning tests (Fig. 1E). Furthermore, no significant differences in extinction learning were detected between wild-type and homo mutant mice (Additional file 1 and Additional file 2: Fig. S1D). In the rotarod test, motor function was partially impaired, but gross motor coordination was not affected, and no anxiety-like behavior was detected in the elevated plus maze test, sociability test, or tail suspension test (Additional file 1 and Additional file 4: Fig. S3A‒D). The het mice were comparable with the wild-type mice even though all the behavioral tests were performed with another batch of mice (Additional file 1 and Additional file 4: Fig. S3E‒G, and Additional file 1 and Additional file 5: Fig. S4A‒C).

### Mutation causes ErbB4-PSD95 dissociations and ErbB4 conformational abnormalities

**Fig. 2:**
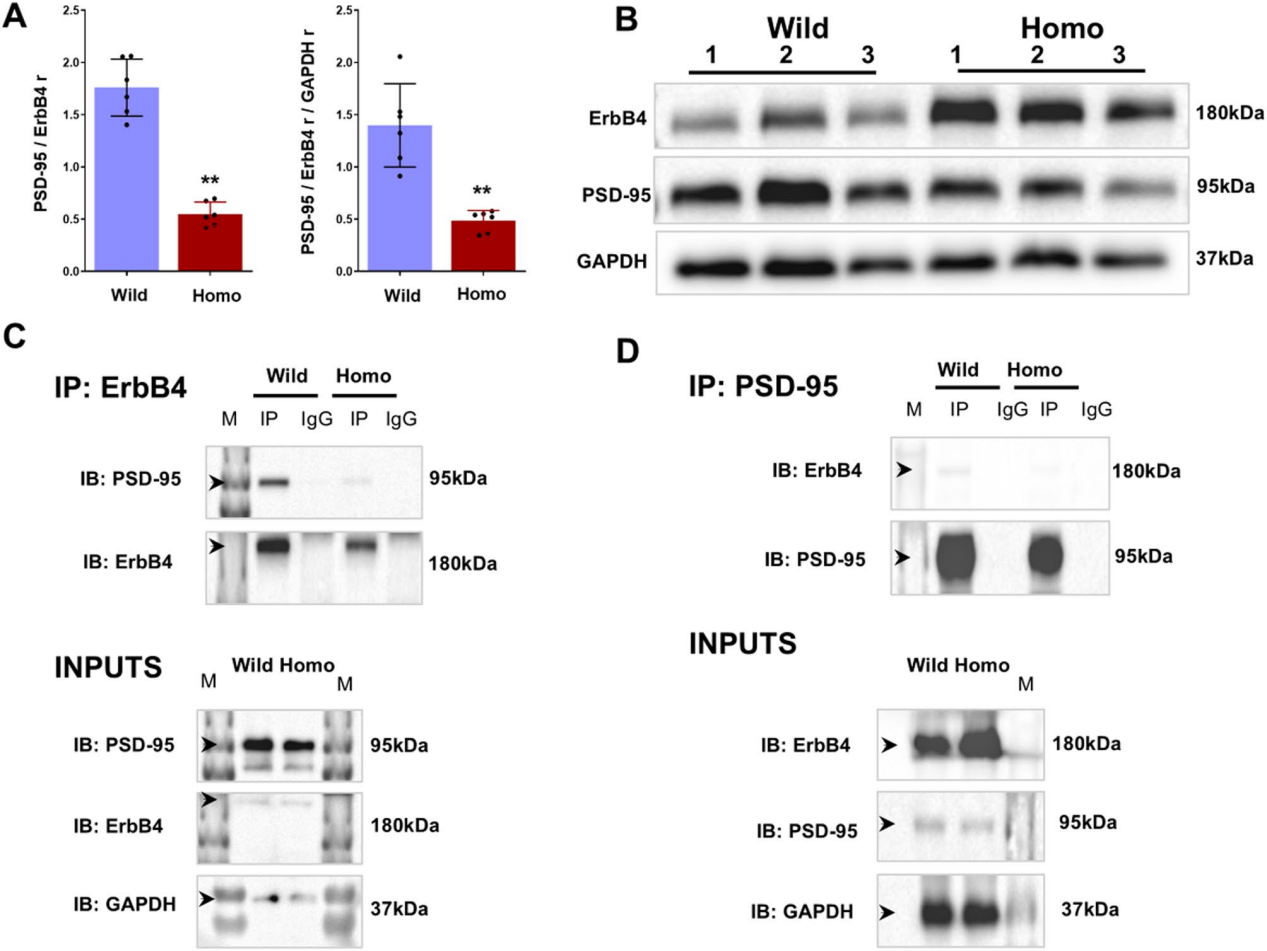
ErbB4 and PSD-95 interaction analysis. Both ErbB4 and PSD-95 were checked in immunoblotting experiments using a single membrane. The association of both proteins (**A**, **B**) were compromised (Student’s t-test, *p* < 0.01, 45 ug protein used, *N* = 3 and *n* = 6). In coimmunoprecipitation experiment results, the ErbB4 antibody was initially used in immunoprecipitation step to catch PSD-95 as a target protein (**C**) and then in reciprocal coimmunoprecipitation experiment, the PSD-95 antibody was used in immunoprecipitation step to catch ErbB4 (**D**). Each coimmunoprecipitation confirms that ErbB4 is not interacting with PSD-95 protein in homo mutant mice. Black color arrows show protein location in **C**,** D**. IP and igG groups = 2000 µg protein and input groups = 45 µg protein used, and *N* = 3 in all groups. N = number of mice, n = number of samples, * = *p* < 0.05, ** = *p* < 0.01

**Fig. 3:**
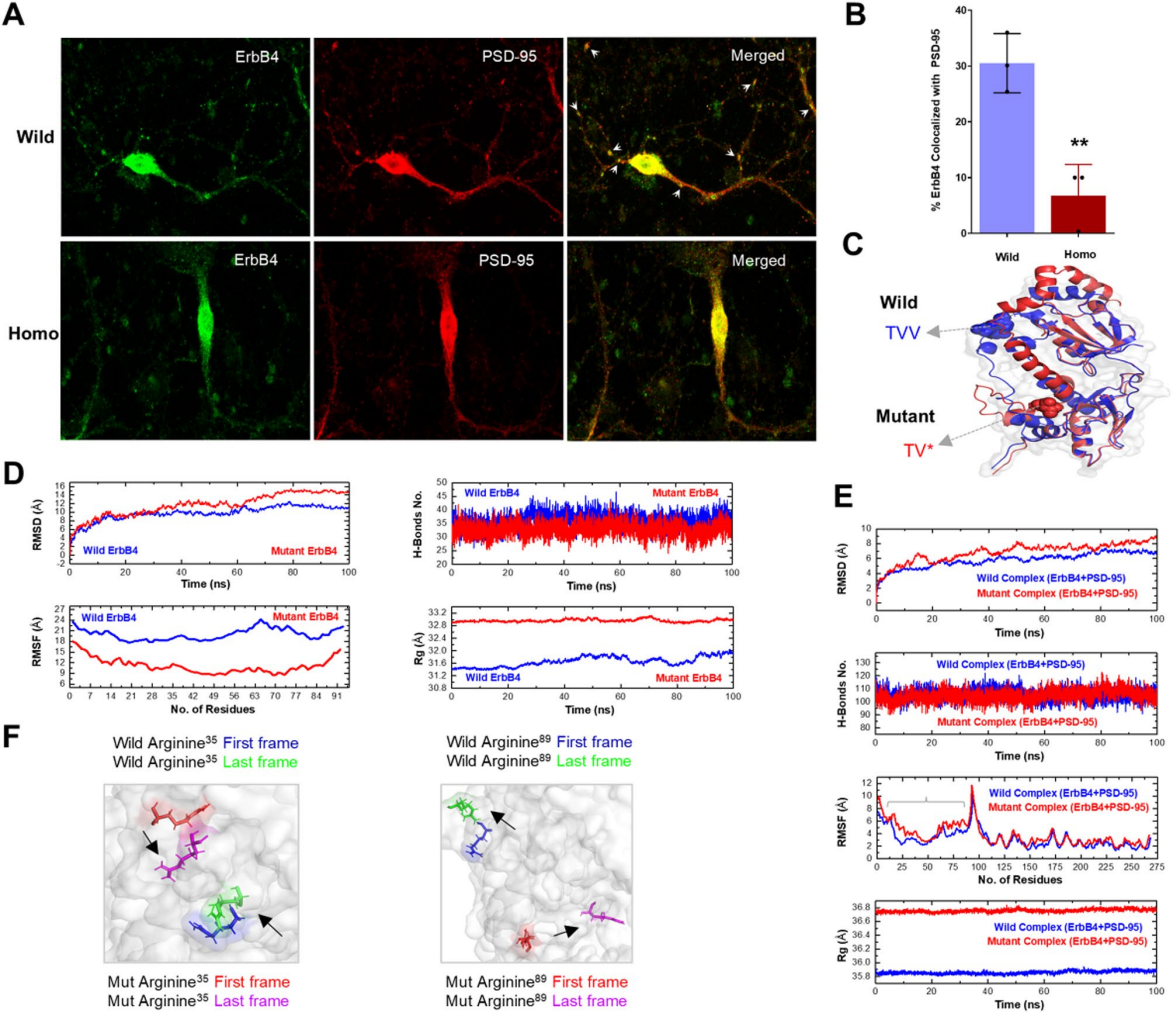
Mutation cause structural deformities in ErbB4 that affects its affinity of binding with PSD-95. ErbB4-PSD-95 colocalization is lost in homo mice cortical primary neuronal culture (**A**). Representative cropped immunofluorescence images (35. 3 μm) shows the ErbB4 (green) is colocalizing with PSD-95 (red) in day 10 primary cortical neurons dendrites. In wild-type neurons, the ErbB4 and PSD-95 signals display significant overlap (yellow color in merged image), indicating colocalization. In mutant ErbB4 and PSD-95 lacks colocalization. White color arrows in zoom merged images shows colocalizing ErbB4 and PSD-95 in wild-type. The percent ErbB4 colocalization (**B**) shows sharp reduction in homo condition (Student’s t-test, *p* < 0.01, *n* = 3). The mutation affected the protein confirmation which was observed in the superimposed docked wild-type (blue color) and mutant ErbB4 (red color) complexes with PSD-95. The TVV motif is shown as spheres (**C**). Post simulation analysis (**D**, **E**) further clarify that mutation causes obvious structural perturbation in ErbB4 alone (apo state) and also drastically effected their binding pattern with PSD-95 protein (holo state). ErbB4 C-terminus tail region residues that showed high flexibility in simulation are marked with light gray color brace in holo state RMSF graph. The position of Arginine 35 (Arg^35^) and Arginine 89 (Arg^89^) of simulated complexes are shown using the superimposed sturctures. First frame (simulation at 0 ns) and last frame (simulation at 100 ns) are considered (**F**) which highlights distinct directional movement during simulation in wild and mutant complexes. Arg^35^ and Arg^89^ are only selected on the basis of their nearby and faraway distance from real mutation position. Fig. **F** is drawn from a single simulation data while **D** and **E** graphs represent mean values obtained from triplicates simulation. n = number of samples, ns = nanosecond, Arg = Arginine, RMSD = Root mean square deviation, RMSF = root mean square fluctuation, No. = number, H = hydrogen, Rg = radius of gyration and ** = *p* < 0.01

The ErbB4-TVV motif is important for the ErbB4-PSD-95 interaction [16, 17], and this protein association is specifically impaired in SCZ [18]. We therefore investigated the effects of ErbB4-TVV mutation on the ErbB4-PSD-95 association in our mouse model. We first performed simple immunoblotting to quantify ErbB4 and PSD-95 and found that the ratio of PSD-95/ErbB4 was highly decreased in the homo mutant, even after GAPDH normalization, as shown by a reduction in the PSD-95/ErbB4 ratio/GAPDH ratio (Fig. 2A, B). This clearly shows that the association of ErbB4 with PSD-95 is affected. We further confirmed this association via reciprocal coimmunoprecipitation experiments. We used an ErbB4 antibody to pull down PSD-95 and found that compared with the wild-type protein, PSD-95 was not detected in homo mutant mice (Fig. 2C). Conversely, when the PSD-95 antibody was used for immunoprecipitation, ErbB4 was also lost in the homo mutant (Fig. 2D). However, PSD-95, ErbB4 and GAPDH were detected in both the wild-type and homo mice groups input internal control groups which confirming the results. ErbB4-PSD-95 dissociation was further confirmed via immunofluorescence staining of day 10 primary neuronal cultures, which revealed that the green signal (ErbB4) did not overlap with the red signal (PSD-95), suggesting that PSD-95 does not colocalize with ErbB4 in the homo mutant (Fig. 3A, B).

This dissociation is possibly caused by a global structural perturbation in the ErbB4 C-terminus due to mutation. To verify this, we performed docking and MD simulation analyses considering the best predicted protein model (See Additional file 1: Table. S1, and Additional file 1 and Additional file 3: Fig. S2). Docking results revealed that the deletion of the last valine “V” from the TVV motif of ErbB4 in mice caused drastic global protein confirmation changes in ErbB4 and affected the binding pattern with PSD-95 (Fig. 3C). The mutant complex has more positive binding affinity and dissociation constant scores, which indicates poor bonding strength (Table 1). Interestingly, although the TVV motif residues are not directly involved in the interaction with PSD-95 (Additional file 1 and Additional file 6: Fig. S5), they are critical for maintaining the structural integrity of the ErbB4 tail region and this structural integrity maintains the binding affinity of ErbB4 to PSD-95. Both the apo (ErbB4 alone) and holo (ErbB4 + PSD-95 complex) state simulation results further confirmed the docking results. In the apo state, as compared to wild-type, the mutant protein was less stable, as indicated by high RMSD values, more rigid, i.e., had lower RMSF values, lost compactness which is indicated by high Rg values and lost natural hydrogen bonding pattern (Fig. 3D). This clearly shows the mutation disrupts the ErbB4 structural behavior. In holo state, the wild-type complex maintained conformational stability and had lower RMSD values than the mutant complex. The mutant complex was found more flexible i.e., had high RMSF values than that of the wild-type complex (Fig. 3E). This indicates that ErbB4 binding with PSD-95 is sensitive to structural flexibility and stability of the tail region of ErbB4, and this pattern is lost due to mutation in ErbB4. Furthermore, the pattern of hydrogen bonding consistency in the beginning (0–60 ns) time is reduced in the mutant complex, which shows the atomic level interaction that is important for binding is lost. Similarly, the mutant complex also lost structural compactness i.e., mutant complex is less compact and has high Rg values (Fig. 3E). All these structural and bonding pattern abnormalities sharply affected the dynamics movement of the complex during simulation (Fig. 3F). These findings highlight the rigidity and decompaction of ErbB4 due to mutation may impair ErbB4’s ability to either recruit or stabilize synaptic proteins and so contributing to synaptic molecular dysfunction.

**Table 1:** Predicted binding affinity and dissociation constant scores predicted by PRODIGY

Parameters	Wild Complex	Mutant Complex
	(ErbB4 + PSD-95)	(Mut ErbB4 + PSD-95)
Binding affinity (ΔG, kcal mol⁻¹)	-15.7	-14.1
Dissociation constant (K_D_, M)	3.3e^− 12^	4.6e^− 11^

### ErbB4 mutation disrupts key postsynaptic proteins

**Fig. 4:**
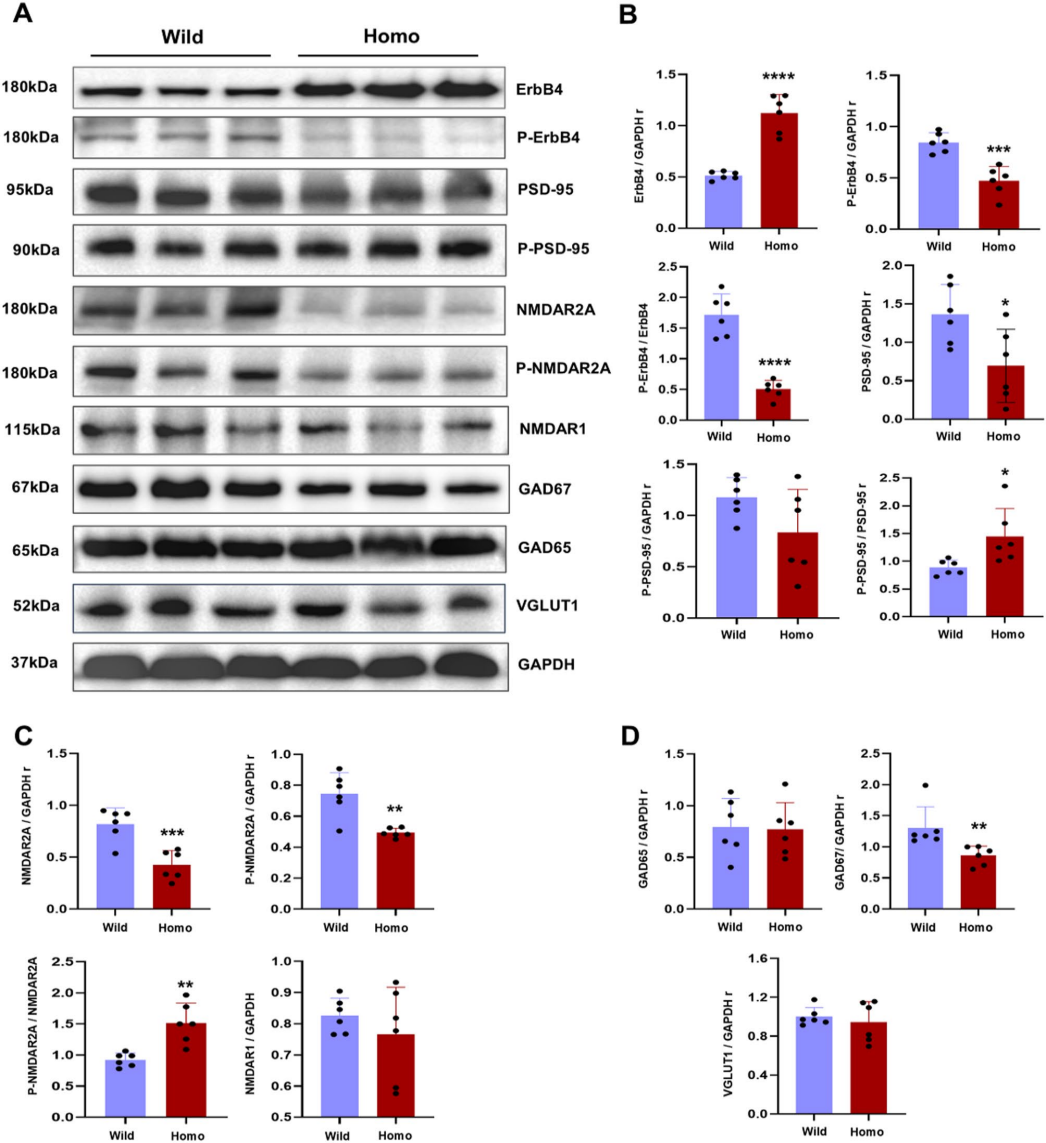
ErbB4 mutation disrupts key proteins of postsynaptic signaling. ErbB4 is increased in homo mice while phosphorylated ErbB4 is decreased and less activated compared to abundance of total protein amount (**A**, **B**, Student’s t-test, *p* < 0.0001, *p* < 0.001, and *p* < 0.0001 respectively). In contrast, the total amount of PSD-95 is decreased (Student’s t-test, *p* < 0.05), but the remaining pool is highly activated in mutant mice (**A**, **B**, Student’s t-test, *p* < 0.05). Mutation also caused hypofunction of NMDAR2A subunit (**A**, **C**). NMDAR2A is highly decreased (Student’s t-test, *p* < 0.001) but hyperactivated in mutant homo mice (Student’s t-test, *p* < 0.01). In contrast, NMDAR1 subunit was not affected which highlights the mutation caused NMDAR subunit specific defects. The GABAergic system is also selectively disrupted (**A**, **D**). GAD65 a global brain GABAergic maker and VGLUT1 were not affected (Student’s t-test, *p* > 0.05) but the GAD67 isoform is specifically highly decreased (Student’s t-test, *p* < 0.01) in mutant homo mice. 60 µg protein, *N* = 3 and *n* = 6 in all experiments, * = *p* < 0.05, ** = *p* < 0.01, *** = *p* < 0.001, **** = *p* < 0.0001. * = *p* < 0.05, ** = *p* < 0.01, *** = *p* < 0.001, **** = *p* < 0.0001, N = number of mice, and n = number of samples

To test whether the dissociation of ErbB4 from PSD-95 also affects its individual protein expression and activation, we performed multiple western blotting experiments and observed significant alterations. ErbB4 was significantly highly expressed, and p-ErbB4 was decreased (Fig. 4A, B). The increase in ErbB4 possibly suggests its potential stabilization mechanism or degradation, which leads to decreased activation of ErbB4. Interestingly, PSD-95 expression and activation were affected in the opposite way, such that total PSD-95 was decreased and the ratio of p-PSD-95/PSD-95 was increased (Fig. 4A, B). Given that ErbB4 is the key synaptic protein that also regulates N-methyl-D-aspartate receptor (NMDAR) function [17, 44]. The mutant mice presented subunit-specific defects in NMDAR expression. NMDAR2A and p-NMDAR2A were noticeably decreased, whereas NMDAR1 remained unaffected (Fig. 4A, C). However, similar to the p-PSD-95/PSD-95 ratio, the p-NMDAR2A/NMDAR2A ratio also increased, indicating that the remaining NMDAR2A and PSD-95 pools are sensitive to phosphorylation. Furthermore, the mutation selectively influenced the GABAergic system by reducing GAD67 expression but not GAD65 expression, while glutamatergic VGLUT1 expression was also not affected (Fig. 4A, D).

## Discussion

In summary, we report the initial findings of a novel zygotic ErbB4 knock-in mouse model. We found that deletion of the terminal valine “V” from the TVV motif of ErbB4 causes the dissociation of ErbB4 from PSD-95 in mice and disrupts the expression and activation of ErbB4 and PSD-95. We computationally explored that this deletion induces a drastic global structural perturbation that makes the ErbB4 C-terminus more rigid and less stable, which impairs its ability to bind with PSD-95. In particular, mutant homo mice further exhibit motor impairments, hypersensitivity and spatial memory defects and thus mirror core SCZ behavioral abnormalities. Besides, the C-terminal tail region of ErbB4 directly interacts with PSD-95 and facilitates NMDAR clustering and regulation [11]. We found that homo mutant mice exhibit subunit-specific defects in NMDAR expression. Both the expression and activation of NMDAR2A were noticeably decreased, whereas those of NMDAR1 remained unaffected. Given that ErbB4 is also involved in modulating both excitatory and inhibitory synaptic functions [22]. We observed a selective reduction in GAD67 expression in homo mutant mice which highlights the preferential vulnerability of the GABAergic system.

The TVV motif of the ErbB4-C-terminus is important for maintaining the binding affinity of the ErbB4-C-terminus for PTZ domain-containing proteins such as PSD-95, PSD-93, and SAP-102 [16, 17]. This motif enables ErbB4 to exhibit diverse signaling patterns at the postsynaptic level. For example, ErbB4 forms a homodimer with another ErbB4 [17] and enables ErbB4 to cross-talk with the NMDAR with the help of PSD-95 [16]. PSD-95 protein complexes have several genes that have been reported in SCZ and abnormalities in these complexes also cause schizophrenia-related behavioral phenotypes [45, 46]. The association of ErbB4 and PSD-95 is further critical for neuronal differentiation [47], NMDAR coupling and influences synaptic plasticity [48, 49]. Alterations in this pathway can cause synaptic dysfunction and structural deficits contributing to the broader pathological synaptic phenotype observed in SCZ [50, 51].

Furthermore, high but less activated ErbB4, possibly highlights a compensatory response to less activation of ErbB4 or that the high expression of ErbB4 is due to the altered association of ErbB4 with PSD-95 [18]. In contrast, phosphorylation of PSD-95 modulates its binding affinity for interacting proteins, either enhancing or inhibiting their interactions [52, 53], regulating synaptic strength [54], and destabilizing postsynaptic density [55]. Therefore, the hyperactivated PSD-95 may represent a failed compensatory response to synaptic deficits caused by mutation, which aims to maintain the ErbB4-PSD-95 interaction (Fig. 4A, B). Overall, these findings align with previous reports showing disrupted ErbB4 and PSD-95 expression in patients with SCZ [18, 56–58].

There is no prior explanation regarding how the TVV motif maintains the structural integrity of the ErbB4 C-terminus. We computationally explained that terminal “V” deletion from the TVV motif caused structural deformities in both the apo (ErbB4 alone) and holo (ErbB4 + PSD-95) forms (Fig. 3C-F). Although the deleted “V” itself may not directly participate in binding with PSD-95, as shown by interaction analysis (Additional files 6: Fig. S5), its absence appears to compromise the structural alignment of the ErbB4 C-terminus, terminal tail movement (Fig. 3C), and kinase activity (less activation, Fig. 4A, B). Our multiple post-simulation structural analyses further support this idea (Fig. 3D-F). The differences in the apo and holo based structural dynamics reported here are commonly observed and arise from the stabilizing effects of ligand binding [59]. These structural perturbations result in the deletion of terminal “V” effects not only at the docking site but also in conformational fluctuations in neighboring residues of the ErbB4 C-terminus, potentially altering the functional dynamics of the ErbB4-C-terminus and thus weakening the interaction interface of the complex (ErbB4 + PSD-95), which is consistent with the idea that the TVV motif is important for protein trafficking and protein‒protein interactions [16, 60].

The ErbB4-PSD-95 association is considerably enhanced in human SCZ patients [18]. Studies in animal models also provide indirect evidence that the ErbB4‒PSD-95 association influences behaviors relevant to SCZ. For example, chronic NMDAR blockade in rats increases ErbB4-PSD-95 and ErbB4-NMDAR interactions and leads to decreased locomotor activity, reduced exploration behavior, and elevated startle magnitudes [49]. Conversely, a reduction in the ErbB4-PSD-95 association has been reported in β-site APP-cleaving enzyme 1 (BACE1)-null mice, which also exhibit behavioral abnormalities, including prepulse inhibition impairments, social defects, and cognitive deficits [61]. These findings show that both hypo- and hyper-ErbB4-PSD-95 association defects converge at SCZ-related phenotypes; thus, homeostasis in ErbB4-PSD-95 is important for normal synaptic biochemical functions. Additionally, NMDAR signaling is known to regulate GAD67 expression via excitation‒transcription coupling [62–64]. Thus, NMDAR hypofunction can lead to the downregulation of GAD67 expression [65, 66] and impair GABAergic neurotransmission. Both GAD67 downregulation and NMDAR2A hypofunction observed in homo mice are considered key biochemical features of SCZ [64, 67], and their hypofunction contributes to inhibitory deficits that further impair E/I balance, another known feature of SCZ pathophysiology [64, 65, 68, 69]. Overall, these findings highlight the major role of the ErbB4-PSD-95-NMDAR axis in the disruption of inhibitory transmission and SCZ-related behavioral phenotypes.

This new genetic ErbB4 mouse model has the potential to provide an in-depth understanding of schizophrenia pathophysiology and can be used in therapeutic studies on SCZ specifically and in other psychiatric diseases. We claim this for five possible reasons: (i) this model has core biochemical and behavioral features of SCZ pathophysiology [65, 69]; (ii) the ErbB4-PSD-95-NMDAR axis disruption in our model represents both GABAergic and glutamatergic abnormalities that can lay the groundwork for therapeutic studies [70]; (iii) ErbB4 is a risk gene for SCZ [8, 9] and some other psychiatric diseases [4–7, 71], but involved molecular pattern and relevant behavioral conseqeunces in these diseases that possibly affecting due to ErbB4-PSD-95 dissociation have not been explored; (iv) the proteins altered in our model can also serve as biomarkers for testing drug efficacy; and (v) it can be a suitable model for screening candidate compounds that modulate two protein interactions, such as roscovitine, which reduces ErbB4-PSD-95 associations [72].

However, the present study has several limitations. Our findings although provide insights into the involvement of the GABAergic system in the observed behavioral phenotypes, but we did not explore the potential developmental deficits in the GABAergic system that possibly contribute to these behavioral deficits. This needs further investigation to explore whether these behavioral deficits arise from early developmental abnormalities or later functional deficits. Another limitation is that there are no rescue experiments in the present study which limits our understanding to establish causality, as restoring functions in this model would further strengthen mechanistic conclusions. Third, we restricted our study to only male mice which limits us to generalize sex-specific effects. Finally, our study lacks deeper functional correlation experiments, such as electrophysiological recordings and calcium imaging. Addressing these limitations in future is important to get more comprehensive understanding of observed effects.

## Conclusion

In conclusion, we developed a novel mouse model by deleting the last valine “V” from the TVV motif of the ErbB4 gene which caused confirmational abnormalities in the ErbB4 C-terminus, compromises its association with PSD-95, and causes motor, sensory, and memory deficits in homozygous condition. The mutation further disturbs the expression and activation of ErbB4 and PSD-95, caused NMDAR2A subunit specific hypofunction, and selective defects in the GABAergic system. We believe this novel ErbB4 mutant mouse is a sensible model that exhibits both behavioral and biochemical defects that mimic SCZ pathophysiology in humans and can be used in future studies for understanding SCZ pathophysiology and therapeutic drugs screening.

## Electronic supplementary material

Below is the link to the electronic supplementary material.

Supplementary Material 1

Supplementary Material 2

Supplementary Material 3

Supplementary Material 4

Supplementary Material 5

Supplementary Material 6

Supplementary Material 7

## Data Availability

Data is provided within the manuscript or supplementary information files.

## References

[1] Fazzari P, Paternain AV, Valiente M, Pla R, Luján R, Lloyd K, et al. Control of cortical GABA circuitry development by Nrg1 and ErbB4 signalling. Nature. 2010;464(7293):1376–80. 20393464 10.1038/nature08928

[2] Dabbah-Assadi F, Alon D, Golani I, Doron R, Kremer I, Beloosesky R, Shamir A. The influence of immune activation at early vs late gestation on fetal NRG1-ErbB4 expression and behavior in juvenile and adult mice offspring. Brain Behav Immun. 2019;79:207–15. 30738182 10.1016/j.bbi.2019.02.002

[3] Tian J, Geng F, Gao F, Chen Y-H, Liu J-H, Wu J-L, et al. Down-regulation of neuregulin1/ErbB4 signaling in the hippocampus is critical for learning and memory. Mol Neurobiol. 2017;54:3976–87. 27295274 10.1007/s12035-016-9956-5

[4] Luykx JJ, Vinkers CH, Bakker SC, Visser WF, Van Boxmeer L, Strengman E, et al. A common variant in ERBB4 regulates GABA concentrations in human cerebrospinal fluid. Neuropsychopharmacology. 2012;37(9):2088–92. 22549119 10.1038/npp.2012.57 PMC3398713

[5] Hyder Z, Van Paesschen W, Sabir A, Sansbury FH, Burke KB, Khan N, et al. ERBB4 exonic deletions on chromosome 2q34 in patients with intellectual disability or epilepsy. Eur J Hum Genet. 2021;29(9):1377–83. 33603162 10.1038/s41431-021-00815-y PMC8440581

[6] Cai Y, Peng Z, He Q, Sun P. Behavioral variant frontotemporal dementia associated with GRN and ErbB4 gene mutations: a case report and literature review. BMC Med Genom. 2024;17(1):43. 10.1186/s12920-024-01819-5 PMC10829211 38291418

[7] Kasnauskiene J, Ciuladaite Z, Preiksaitiene E, Utkus A, Peciulyte A, Kučinskas V. A new single gene deletion on 2q34: ERBB4 is associated with intellectual disability. Am J Med Genet Part A. 2013;161(6):1487–90. 10.1002/ajmg.a.35911 23633123

[8] Chen P, Chen J, Huang K, Ji W, Wang T, Li T, et al. Analysis of association between common SNPs in ErbB4 and bipolar affective disorder, major depressive disorder and schizophrenia in the Han Chinese population. Prog Neuropsychopharmacol Biol Psychiatry. 2012;36(1):17–21. 21993442 10.1016/j.pnpbp.2011.09.011

[9] Mei L, Nave K-A. Neuregulin-ERBB signaling in the nervous system and neuropsychiatric diseases. Neuron. 2014;83(1):27–49. 24991953 10.1016/j.neuron.2014.06.007 PMC4189115

[10] Feng Y, Cheng D, Zhang C, Li Y, Zhang Z, Wang J, Feng X. Association between ErbB4 single nucleotide polymorphisms and susceptibility to schizophrenia: A meta-analysis of case-control studies. Medicine. 2017;96(8):e5920. 28225484 10.1097/MD.0000000000005920 PMC5569411

[11] Greenwood TA, Light GA, Swerdlow NR, Radant AD, Braff DL. Association analysis of 94 candidate genes and schizophrenia-related endophenotypes. PLoS ONE. 2012;7(1):e29630. 22253750 10.1371/journal.pone.0029630 PMC3258248

[12] Wang H, Liu F, Chen W, Sun X, Cui W, Dong Z, et al. Genetic recovery of ErbB4 in adulthood partially restores brain functions in null mice. Proc Natl Acad Sci. 2018;115(51):13105–10. 30498032 10.1073/pnas.1811287115 PMC6304932

[13] Del Pino I, García-Frigola C, Dehorter N, Brotons-Mas JR, Alvarez-Salvado E, de Lagrán MM, et al. Erbb4 deletion from fast-spiking interneurons causes schizophrenia-like phenotypes. Neuron. 2013;79(6):1152–68. 24050403 10.1016/j.neuron.2013.07.010

[14] Wen L, Lu Y-S, Zhu X-H, Li X-M, Woo R-S, Chen Y-J et al. Neuregulin 1 regulates pyramidal neuron activity via ErbB4 in parvalbumin-positive interneurons. Proceedings of the National Academy of Sciences. 2010;107(3):1211-6. 10.1073/pnas.0910302107 PMC2824309 20080551

[15] Skirzewski M, Cronin ME, Murphy R, Fobbs W, Kravitz AV, Buonanno A. ErbB4 null mice display altered mesocorticolimbic and nigrostriatal dopamine levels as well as deficits in cognitive and motivational behaviors. Eneuro. 2020;7(3). 10.1523/ENEURO.0395-19.2020 PMC7242816 32354758

[16] Garcia RA, Vasudevan K, Buonanno A. The neuregulin receptor ErbB-4 interacts with PDZ-containing proteins at neuronal synapses. Proceedings of the National Academy of Sciences. 2000;97(7):3596– 601. 10.1073/pnas.070042497 PMC16285 10725395

[17] Huang YZ, Won S, Ali DW, Wang Q, Tanowitz M, Du QS, et al. Regulation of neuregulin signaling by PSD-95 interacting with ErbB4 at CNS synapses. Neuron. 2000;26(2):443–55. 10839362 10.1016/s0896-6273(00)81176-9

[18] Hahn C-G, Wang H-Y, Cho D-S, Talbot K, Gur RE, Berrettini WH, et al. Altered neuregulin 1–erbB4 signaling contributes to NMDA >receptor hypofunction in schizophrenia. Nat Med. 2006;12(7):824–8. 16767099 10.1038/nm1418

[19] Li B, Woo R-S, Mei L, Malinow R. The neuregulin-1 receptor erbB4 controls glutamatergic synapse maturation and plasticity. Neuron. 2007;54(4):583–97. 17521571 10.1016/j.neuron.2007.03.028 PMC2031848

[20] Lu Y, Sun X-D, Hou F-Q, Bi L-L, Yin D-M, Liu F, et al. Maintenance of GABAergic activity by neuregulin 1-ErbB4 in amygdala for fear memory. Neuron. 2014;84(4):835–46. 25451196 10.1016/j.neuron.2014.09.029

[21] Tamura H, Kawata M, Hamaguchi S, Ishikawa Y, Shiosaka S. Processing of neuregulin-1 by neuropsin regulates GABAergic neuron to control neural plasticity of the mouse hippocampus. J Neurosci. 2012;32(37):12657–72. 22972991 10.1523/JNEUROSCI.2542-12.2012 PMC6703812

[22] Chen Y-J, Zhang M, Yin D-M, Wen L, Ting A, Wang P, et al. ErbB4 in parvalbumin-positive interneurons is critical for neuregulin 1 regulation of long-term potentiation. Proc Natl Acad Sci. 2010;107(50):21818–23. 21106764 10.1073/pnas.1010669107 PMC3003111

[23] Bi L-L, Sun X-D, Zhang J, Lu Y-S, Chen Y-H, Wang J, et al. Amygdala NRG1–ErbB4 is critical for the modulation of anxiety-like behaviors. Neuropsychopharmacology. 2015;40(4):974–86. 25308353 10.1038/npp.2014.274 PMC4330511

[24] Chen Y-H, Hu N-Y, Wu D-Y, Bi L-L, Luo Z-Y, Huang L, et al. PV network plasticity mediated by neuregulin1-ErbB4 signalling controls fear extinction. Mol Psychiatry. 2022;27(2):896–906. 34697452 10.1038/s41380-021-01355-z

[25] Ting AK, Chen Y, Wen L, Yin D-M, Shen C, Tao Y, et al. Neuregulin 1 promotes excitatory synapse development and function in GABAergic interneurons. J Neurosci. 2011;31(1):15–25. 21209185 10.1523/JNEUROSCI.2538-10.2011 PMC3078582

[26] Marenco S, van der Geramita M, Barnett AS, Kolachana B, Shen J, et al. Genetic association of ErbB4 and human cortical GABA levels in vivo. J Neurosci. 2011;31(32):11628–32. 21832192 10.1523/JNEUROSCI.1529-11.2011 PMC3159177

[27] Neddens J, Buonanno A. Selective populations of hippocampal interneurons express ErbB4 and their number and distribution is altered in ErbB4 knockout mice. Hippocampus. 2010;20(6):724–44. 19655320 10.1002/hipo.20675 PMC2958210

[28] He G, Wang X-Y, Jia Z, Zhou Z. Characterizing neurotrophic factor-induced synaptic growth in primary mouse neuronal cultures. STAR Protocols. 2022;3(1):101112. 35098164 10.1016/j.xpro.2021.101112 PMC8783201

[29] Schindelin J, Arganda-Carreras I, Frise E, Kaynig V, Longair M, Pietzsch T, et al. Fiji: an open-source platform for biological-image analysis. Nat Methods. 2012;9(7):676–82. 22743772 10.1038/nmeth.2019 PMC3855844

[30] Bolte S, Cordelières FP. A guided tour into subcellular colocalization analysis in light microscopy. J Microsc. 2006;224(3):213–32. 17210054 10.1111/j.1365-2818.2006.01706.x

[31] De Vries SJ, Van Dijk M, Bonvin AM. The HADDOCK web server for data-driven biomolecular Docking. Nat Protoc. 2010;5(5):883–97. 20431534 10.1038/nprot.2010.32

[32] Magrane M, Consortium U. UniProt knowledgebase: a hub of integrated protein data. Database. 2011;2011:bar009. 21447597 10.1093/database/bar009 PMC3070428

[33] Yang J, Zhang Y. I-TASSER server: new development for protein structure and function predictions. Nucleic Acids Res. 2015;43(W1):W174–81. 25883148 10.1093/nar/gkv342 PMC4489253

[34] Abramson J, Adler J, Dunger J, Evans R, Green T, Pritzel A, et al. Accurate structure prediction of biomolecular interactions with alphafold 3. Nature. 2024;630(8016):493–500. 38718835 10.1038/s41586-024-07487-w PMC11168924

[35] Harris RC, Chung E, Coffey RJ. EGF receptor ligands. EGF Receptor Family. 2003:3–14. 10.1016/s0014-4827(02)00105-2 12648462

[36] Walsh T, McClellan JM, McCarthy SE, Addington AM, Pierce SB, Cooper GM, et al. Rare structural variants disrupt multiple genes in neurodevelopmental pathways in schizophrenia. Science. 2008;320(5875):539–43. 18369103 10.1126/science.1155174

[37] Vangone A, Bonvin AM. Contacts-based prediction of binding affinity in protein–protein complexes. elife. 2015;4:e07454. 26193119 10.7554/eLife.07454 PMC4523921

[38] Xue LC, Rodrigues JP, Kastritis PL, Bonvin AM, Vangone A. PRODIGY: a web server for predicting the binding affinity of protein–protein complexes. Bioinformatics. 2016;32(23):3676-8. 27503228 10.1093/bioinformatics/btw514

[39] Salomon-Ferrer R, Gotz AW, Poole D, Le Grand S, Walker RC. Routine microsecond molecular dynamics simulations with AMBER on gpus. 2. Explicit solvent particle mesh Ewald. J Chem Theory Comput. 2013;9(9):3878–88. 26592383 10.1021/ct400314y

[40] Price DJ, Brooks CL III. A modified TIP3P water potential for simulation with Ewald summation. J Chem Phys. 2004;121(20):10096–103. 15549884 10.1063/1.1808117

[41] Meza JC. Steepest descent. Wiley Interdisciplinary Reviews: Comput Stat. 2010;2(6):719–22.

[42] Watowich SJ, Meyer ES, Hagstrom R, Josephs R. A stable, rapidly converging conjugate gradient method for energy minimization. J Comput Chem. 1988;9(6):650–61.

[43] Roe DR, Cheatham TE III. PTRAJ and CPPTRAJ: software for processing and analysis of molecular dynamics trajectory data. J Chem Theory Comput. 2013;9(7):3084–95. 26583988 10.1021/ct400341p

[44] Asede D, Okoh J, Ali S, Doddapaneni D, Bolton MM. Deletion of ErbB4 disrupts synaptic transmission and long-term potentiation of thalamic input to amygdalar medial paracapsular intercalated cells. Front Synaptic Neurosci. 2021;13:697110. 34393751 10.3389/fnsyn.2021.697110 PMC8355744

[45] Matosin N, Fernandez-Enright F, Lum JS, Engel M, Andrews JL, Gassen NC, et al. Molecular evidence of synaptic pathology in the CA1 region in schizophrenia. Npj Schizophrenia. 2016;2(1):1–8. 27430010 10.1038/npjschz.2016.22 PMC4944906

[46] Coley AA, Gao W-J. PSD95: A synaptic protein implicated in schizophrenia or autism? Progress in Neuro-Psychopharmacology and biological psychiatry. 2018;82:187–94. 10.1016/j.pnpbp.2017.11.016 PMC5801047 29169997

[47] Murphy SP, Bielby-Clarke K. Neuregulin signaling in neurons depends on ErbB4 interaction with PSD-95. Brain Res. 2008;1207:32–5. 18374309 10.1016/j.brainres.2008.02.063

[48] Vullhorst D, Mitchell RM, Keating C, Roychowdhury S, Karavanova I, Tao-Cheng J-H, Buonanno A. A negative feedback loop controls NMDA receptor function in cortical interneurons via neuregulin 2/ErbB4 signalling. Nat Commun. 2015;6(1):7222. 26027736 10.1038/ncomms8222 PMC4451617

[49] Li J-T, Feng Y, Su Y-A, Wang X-D, Si T-M. Enhanced interaction among ErbB4, PSD-95 and NMDAR by chronic MK-801 treatment is associated with behavioral abnormalities. Pharmacol Biochem Behav. 2013;108:44–53. 23603030 10.1016/j.pbb.2013.04.008

[50] Coyle JT, Tsai G. NMDA receptor function, neuroplasticity, and the pathophysiology of schizophrenia. Int Rev Neurobiol. 2004;59:491–515. 15006500 10.1016/S0074-7742(04)59019-0

[51] Kristiansen LV, Huerta I, Beneyto M, Meador-Woodruff JH. NMDA receptors and schizophrenia. Curr Opin Pharmacol. 2007;7(1):48–55. 17097347 10.1016/j.coph.2006.08.013

[52] Vistrup-Parry M, Chen X, Johansen TL, Bach S, Buch-Larsen SC, Bartling CR et al. Site-specific phosphorylation of PSD-95 dynamically regulates the postsynaptic density as observed by phase separation. Iscience. 2021;24(11). 10.1016/j.isci.2021.103268 PMC8567388 34761188

[53] Pedersen SW, Albertsen L, Moran GE, Levesque B, Pedersen SB, Bartels L, et al. Site-specific phosphorylation of PSD-95 PDZ domains reveals fine-tuned regulation of protein–protein interactions. ACS Chem Biol. 2017;12(9):2313–23. 28692247 10.1021/acschembio.7b00361 PMC6081957

[54] Kim MJ, Futai K, Jo J, Hayashi Y, Cho K, Sheng M. Synaptic accumulation of PSD-95 and synaptic function regulated by phosphorylation of serine-295 of PSD-95. Neuron. 2007;56(3):488–502. 17988632 10.1016/j.neuron.2007.09.007

[55] Steiner P, Higley MJ, Xu W, Czervionke BL, Malenka RC, Sabatini BL. Destabilization of the postsynaptic density by PSD-95 Serine 73 phosphorylation inhibits spine growth and synaptic plasticity. Neuron. 2008;60(5):788–802. 19081375 10.1016/j.neuron.2008.10.014 PMC2671083

[56] Joshi D, Fullerton JM, Weickert CS. Elevated ErbB4 mRNA is related to interneuron deficit in prefrontal cortex in schizophrenia. J Psychiatr Res. 2014;53:125–32. 24636039 10.1016/j.jpsychires.2014.02.014

[57] Ohnuma T, Kato H, Arai H, Faull RL, McKenna PJ, Emson PC. Gene expression of PSD95 in prefrontal cortex and hippocampus in schizophrenia. NeuroReport. 2000;11(14):3133–7. 11043537 10.1097/00001756-200009280-00019

[58] Catts VS, Derminio DS, Hahn C-G, Weickert CS. Postsynaptic density levels of the NMDA receptor NR1 subunit and PSD-95 protein in prefrontal cortex from people with schizophrenia. Npj Schizophrenia. 2015;1(1):1–8. 10.1038/npjschz.2015.37 PMC4849460 27336043

[59] Bouyain S, Longo PA, Li S, Ferguson KM, Leahy DJ. The extracellular region of ErbB4 adopts a tethered conformation in the absence of ligand. Proceedings of the National Academy of Sciences. 2005;102(42):15024-9. 10.1073/pnas.0507591102 PMC1257738 16203964

[60] Gallo RM, Bryant IN, Mill CP, Kaverman S, Riese DJ. Multiple functional motifs are required for the tumor suppressor activity of a constitutively-active ErbB4 mutant. J cancer Res Therapeutic Oncol. 2013;1(1):10. PMC4002051 24791013

[61] Savonenko A, Melnikova T, Laird F, Stewart K-A, Price D, Wong P. Alteration of BACE1-dependent NRG1/ErbB4 signaling and schizophrenia-like phenotypes in BACE1-null mice. Proc Natl Acad Sci. 2008;105(14):5585–90. 18385378 10.1073/pnas.0710373105 PMC2291091

[62] Kinney JW, Davis CN, Tabarean I, Conti B, Bartfai T, Behrens MM. A specific role for NR2A-containing NMDA receptors in the maintenance of parvalbumin and GAD67 immunoreactivity in cultured interneurons. J Neurosci. 2006;26(5):1604–15. 16452684 10.1523/JNEUROSCI.4722-05.2006 PMC6675509

[63] Curley AA, Eggan SM, Lazarus MS, Huang ZJ, Volk DW, Lewis DA. Role of glutamic acid decarboxylase 67 in regulating cortical parvalbumin and GABA membrane transporter 1 expression: implications for schizophrenia. Neurobiol Dis. 2013;50:179–86. 23103418 10.1016/j.nbd.2012.10.018 PMC3534919

[64] Fujihara K, Miwa H, Kakizaki T, Kaneko R, Mikuni M, Tanahira C, et al. Glutamate decarboxylase 67 deficiency in a subset of GABAergic neurons induces schizophrenia-related phenotypes. Neuropsychopharmacology. 2015;40(10):2475–86. 25904362 10.1038/npp.2015.117 PMC4538341

[65] Cohen SM, Tsien RW, Goff DC, Halassa MM. The impact of NMDA receptor hypofunction on GABAergic neurons in the pathophysiology of schizophrenia. Schizophr Res. 2015;167(1–3):98–107. 25583246 10.1016/j.schres.2014.12.026 PMC4724170

[66] Fujihara K. Beyond the γ-aminobutyric acid hypothesis of schizophrenia. Front Cell Neurosci. 2023;17:1161608. 37168420 10.3389/fncel.2023.1161608 PMC10165250

[67] Nakazawa K, Sapkota K. The origin of NMDA receptor hypofunction in schizophrenia. Pharmacol Ther. 2020;205:107426. 31629007 10.1016/j.pharmthera.2019.107426 PMC6981256

[68] Lányi O, Koleszár B, Schulze Wenning A, Balogh D, Engh MA, Horváth AA, et al. Excitation/inhibition imbalance in schizophrenia: a meta-analysis of inhibitory and excitatory TMS-EMG paradigms. Schizophrenia. 2024;10(1):56. 38879590 10.1038/s41537-024-00476-y PMC11180212

[69] Uliana DL, Lisboa JRF, Gomes FV, Grace AA. The excitatory-inhibitory balance as a target for the development of novel drugs to treat schizophrenia. Biochem Pharmacol. 2024:116298. 10.1016/j.bcp.2024.116298 PMC11410545 38782077

[70] Kruse AO, Bustillo JR. Glutamatergic dysfunction in schizophrenia. Translational Psychiatry. 2022;12(1):500. 36463316 10.1038/s41398-022-02253-w PMC9719533

[71] Takahashi Y, Fukuda Y, Yoshimura J, Toyoda A, Kurppa K, Moritoyo H, et al. ERBB4 mutations that disrupt the neuregulin-ErbB4 pathway cause amyotrophic lateral sclerosis type 19. Am J Hum Genet. 2013;93(5):900–5. 24119685 10.1016/j.ajhg.2013.09.008 PMC3824132

[72] Xie F, Padival M, Siegel RE. Association of PSD-95 with ErbB4 facilitates neuregulin signaling in cerebellar granule neurons in culture. J Neurochem. 2007;100(1):62–72. 17074065 10.1111/j.1471-4159.2006.04182.x

