# Supplementary material for: Exploration of schizophrenia-related behavioral and molecular abnormalities in a mutant mouse model with a mutation in the TVV motif of the ErbB4 gene
Source: Mol Brain. 2025 Oct 9;18:78. doi: 10.1186/s13041-025-01238-2 (PMC12513098; doi:10.1186/s13041-025-01238-2)
Supplement: Supplementary file 1 — Supplementary Material 1 [file 13041_2025_1238_MOESM1_ESM.docx]

**Supplementary figure and table legends**

**Fig. S1** No group differences in extinction learning and retrieval tests**.** Schematic presentation of ErbB4 domains. It consists of a signal peptide (SP) sequence, four domains (I-IV), a stalk region, transmembrane domain (TM), tyrosine Kinase domain (TKD) and a carboxyl-terminal domain that have a TVV sequence that acts as a PTZ binding motif [modified from Ref. 35] (**A)**. The targeting strategy allows the generation of a point mutation knockin (KI) mErbb4 alleles. Last valine encoded by GTG will be deleted in 3’ homology arm by site-directed mutagenesis (**B**). No effect on body weight (**C**) (ANOVA test, p > 0.05, wild: N = 6, het: N = 13, and homo: N = 8). No differences in fear extinction or memory retention (**D**) found between wild-type and homo (Mann-Whitney U-test, p > 0.05, Wild: N = 30, and homo: N = 29. * = p < 0.05, ** = p < 0.01, *** = p < 0.001, **** = p < 0.0001, and N = number of mice.

**Fig. S2** Protein structural models. ErbB4 C-Terminal only and PSD-95 domain 1 and 2 structures were prepared using Alphafold3 and i-Tasser modeling tools. Also see Supp Table. 1 for quality check scores of these models (**A**). Top 6 Docked wild complexes (Wild ErbB4+PSD-95) (**B**). Comparative RMSD analysis graph for wild-type complexes (**C**). Top 6 docked models for mutant complexes (Mutant ErbB4+PSD-95) (**D**). Comparative RMSD analysis graph for mutant docked complexes (**E**). Model that has lowest comparative RMSD score was considered for molecular dynamics simulation. In both cases model 1 was selected. Wild ErbB4 is shown with green color, mutant ErbB4 with brown color and PSD-95 with cyan color

**Fig. S3**

No behavioral defects in motor and anxiety related tests. No defects were found in time spent on rod in rotarod test in mutant mice, all groups time were comparable (**A**) (ANOVA test, p > 0.05). Time in open arms and frequency of entrance (**B**) were also comparable in elevated plus maze (ANOVA test, p > 0.05). The tail suspension test time (**C**) was also non-significant as well as no abnormal normal social behavior (**D**) was observed (ANOVA test, p > 0.05 in both cases). **A**-**D** wild: N = 14, het: N = 30 het and homo: N = 11. Another batch of het and wild-type were again checked in open filed (**E**), elevated plus maze (**F**) and forced swim test (**G**) tests of wild-type, but no behavioral defects observed (Student's t-test, p > 0.05 in all three tests, wild: N = 10 and het: N = 9). * = p < 0.05. * = p < 0.05, ** = p < 0.01, *** = p < 0.001, **** = p < 0.0001, and N = number of mice

**Fig. S4** Het mice have normal behavioral phenotypes**. A-C** shows social exploration, fear conditioning and fear conditioning tests of wild-type and het mice, but no behavioral defects were observed (Student's t-test, p > 0.05 in all three tests, wild: N = 10 and het: N = 9). * = p < 0.05, ** = p < 0.01, *** = p < 0.001, **** = p < 0.0001, and N = number of mice

**Fig. S5** The specific individual amino acids bonding pattern is drastically affected. Docking interaction figures shows that TVV motif amino acids that are represented by Thr^91^, Val^92^, Val^93^ of the ErbB4 are not involved in direct interaction with PSD-95

**Table S1** Quality check analysis of prepared protein structural models. Based on the overall good quality scores bold highlighted and underlined protein models were further considered for molecular docking.  C-score is typically in the range of [-5, 2], where a C-score of a higher value signifies a model. High % of Ramchandran and ERAAT2 scores generally indicates models’ reliability.

| **Tool** | **Protein** | **Model** | **Alphafold3 Score** | **i-Tasser**  **C Score** | **Ramchandran**  **Plot Score %** | | **ERAAT2**  **Score %** | **Ranked by lowest RMSD** |
| --- | --- | --- | --- | --- | --- | --- | --- | --- |
|  |  |  |  |  | **Allowed** | **+ Allowed** |  |  |
| Alphafold3 | ErbB4  wild | 1 | 0.61 | - | 93.4 | 6.6 | 89.15 | 3 |
|  |  | 2 | 0.55 | - | 94.7 | 5.3 | 86.90 | 2 |
|  |  | **3** | 0.41 | - | 90.8 | 6.6 | 94.11 | 1 |
|  | ErbB4  Mutant | 1 | 0.71 | - | 86.7 | 12.0 | 80.48 | 2 |
|  |  | **2** | 0.60 | - | 94.7 | 5.3 | 90.24 | 1 |
|  |  | 3 | 0.60 | - | 96.6 | 4.0 | 83.13 | 3 |
|  | PSD-95 | **1** | 0.51 | - | 88.8 | 9.9 | 97.22 | 2 |
|  |  | 2 | 0.49 | - | 88.7 | 10.6 | 95.67 | 1 |
|  |  | 3 | 0.49 | - | 90.7 | 8.6 | 96.27 | 3 |
| i-Tasser | ErbB4  wild | 1 | - | -3.32 | 31.6 | 56.8 | 76.47 | 3 |
|  |  | 2 | - | -4.24 | 35.5 | 31.6 | 63.52 | 2 |
|  |  | 3 | - | -4.05 | 34.2 | 42.1 | 34.11 | 1 |
|  | ErbB4  Mutant | 1 | - | -4.27 | 24 | 48 | 11.90 | 2 |
|  |  | 2 | - | -4.18 | 90.7 | 8.6 | 54.76 | 1 |
|  |  | 3 | - | -4.29 | 33.3 | 45.3 | 44.04 | 3 |
|  | PSD-95 | 1 | - | -1.63 | 57.6 | 34.4 | 89.22 | 1 |
|  |  | 2 | - | -2.09 | 57.6 | 33.1 | 83.83 | 2 |
|  |  | 3 | - | -1.90 | 57.6 | 34.4 | 82.63 | 3 |
