# Supplementary figures and images for: Exploration of schizophrenia-related behavioral and molecular abnormalities in a mutant mouse model with a mutation in the TVV motif of the ErbB4 gene
Source: Mol Brain. 2025 Oct 9;18:78. doi: 10.1186/s13041-025-01238-2 (PMC12513098; doi:10.1186/s13041-025-01238-2)

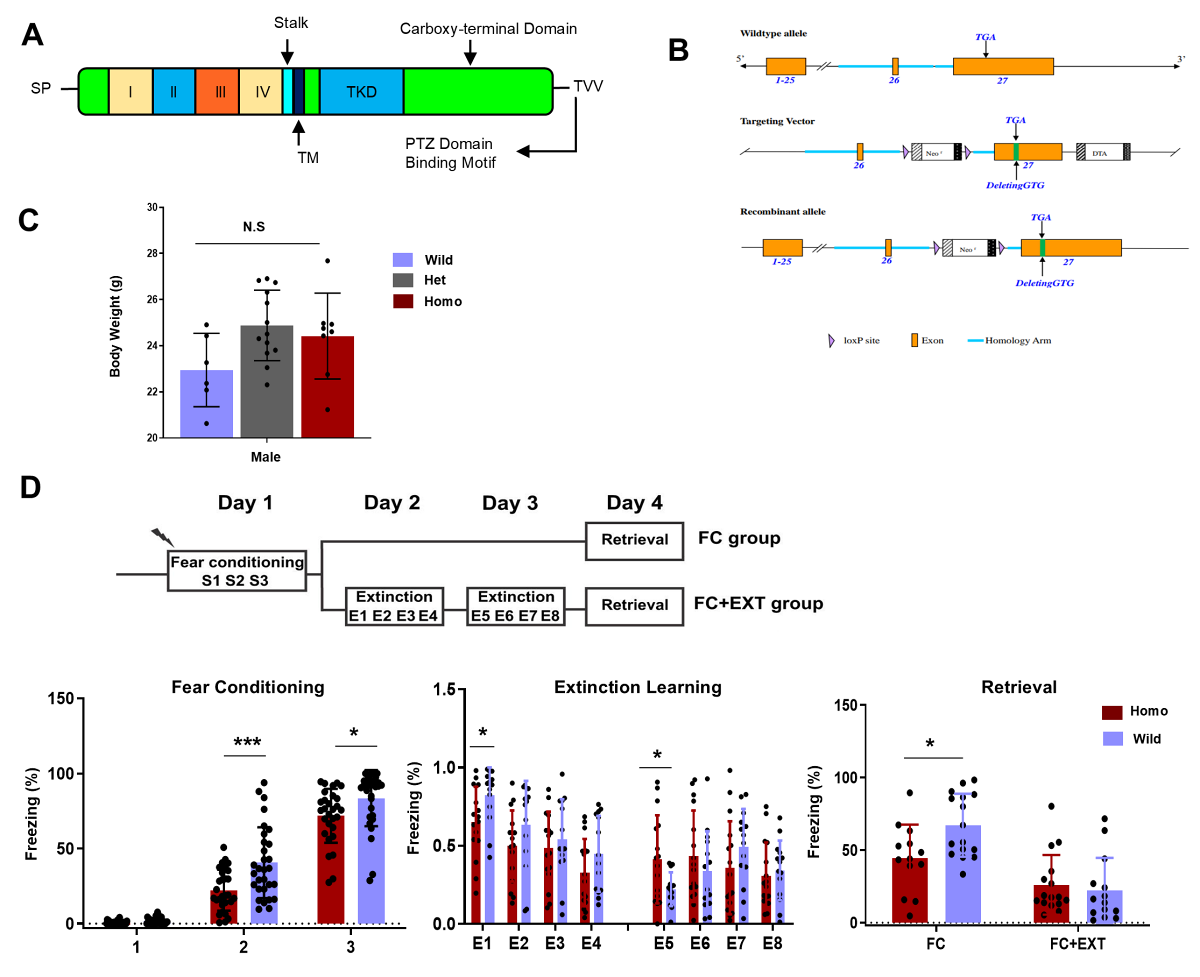

Supplement: Supplementary file 3 — Supplementary Material 3 [file 13041_2025_1238_MOESM3_ESM.tif]

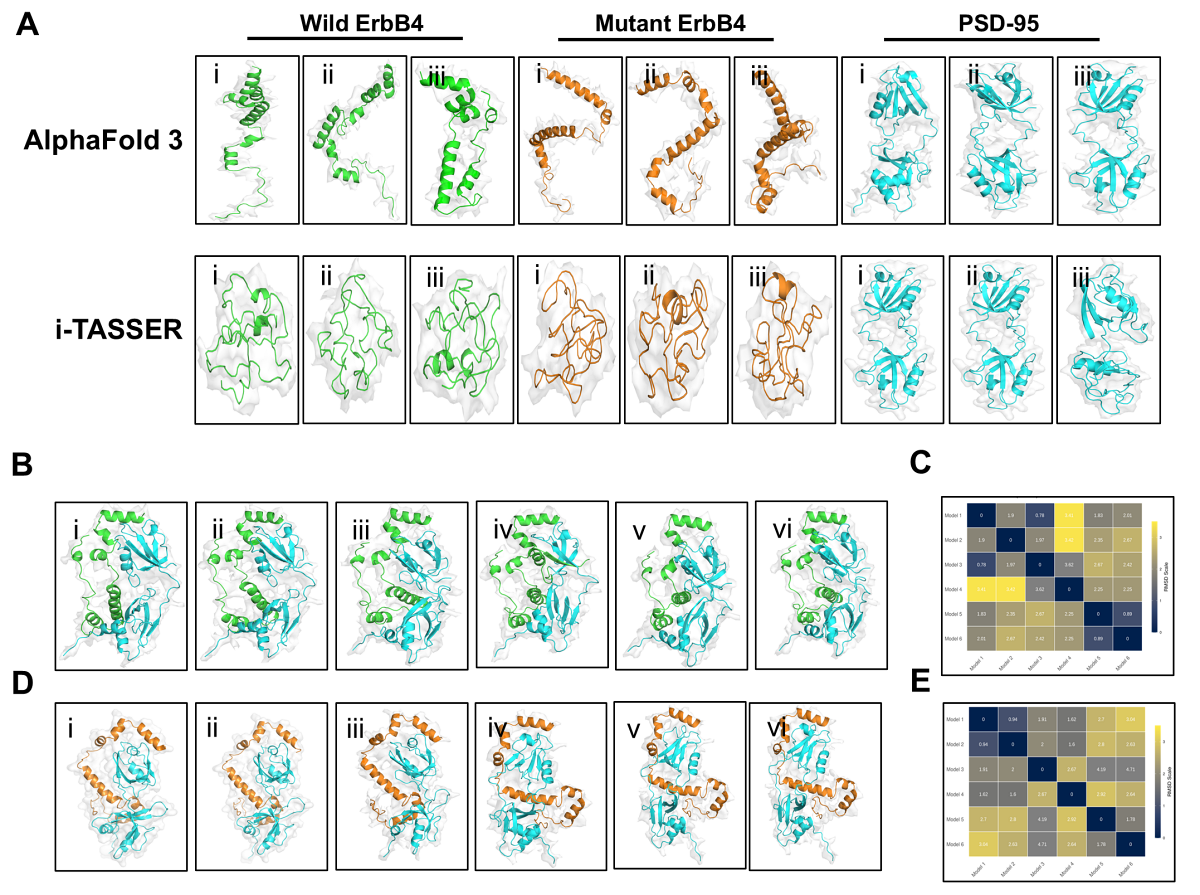

Supplement: Supplementary file 4 — Supplementary Material 4 [file 13041_2025_1238_MOESM4_ESM.tif]

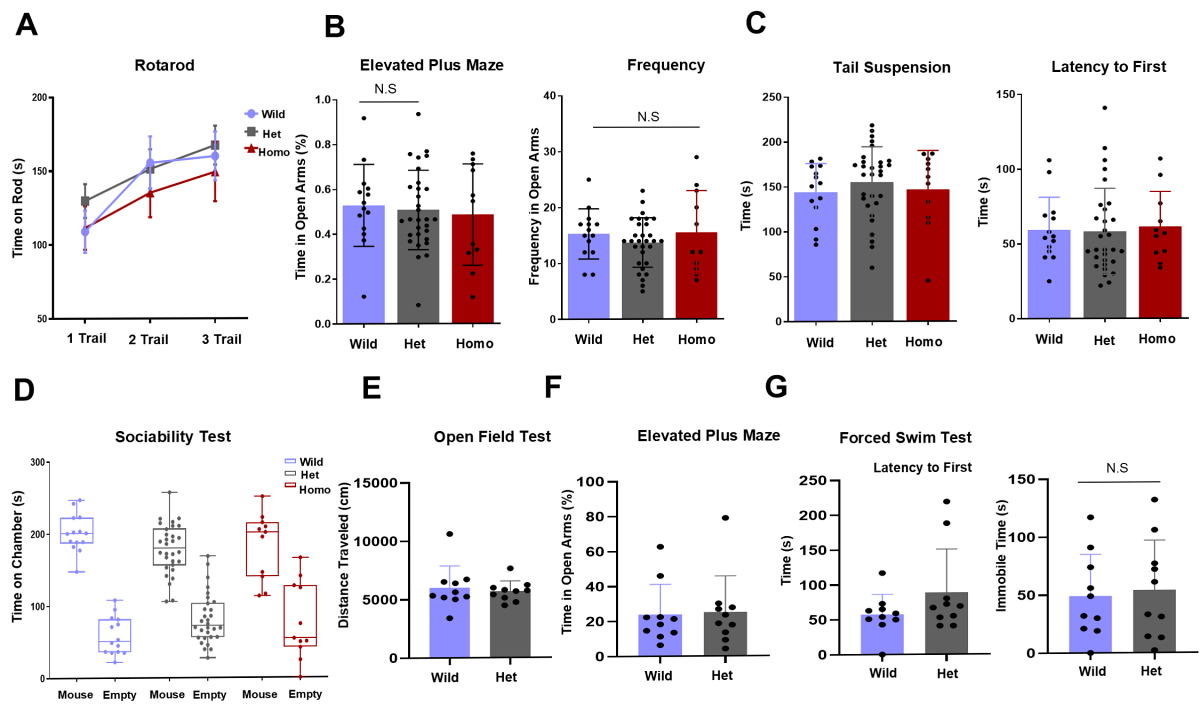

Supplement: Supplementary file 5 — Supplementary Material 5 [file 13041_2025_1238_MOESM5_ESM.tif]

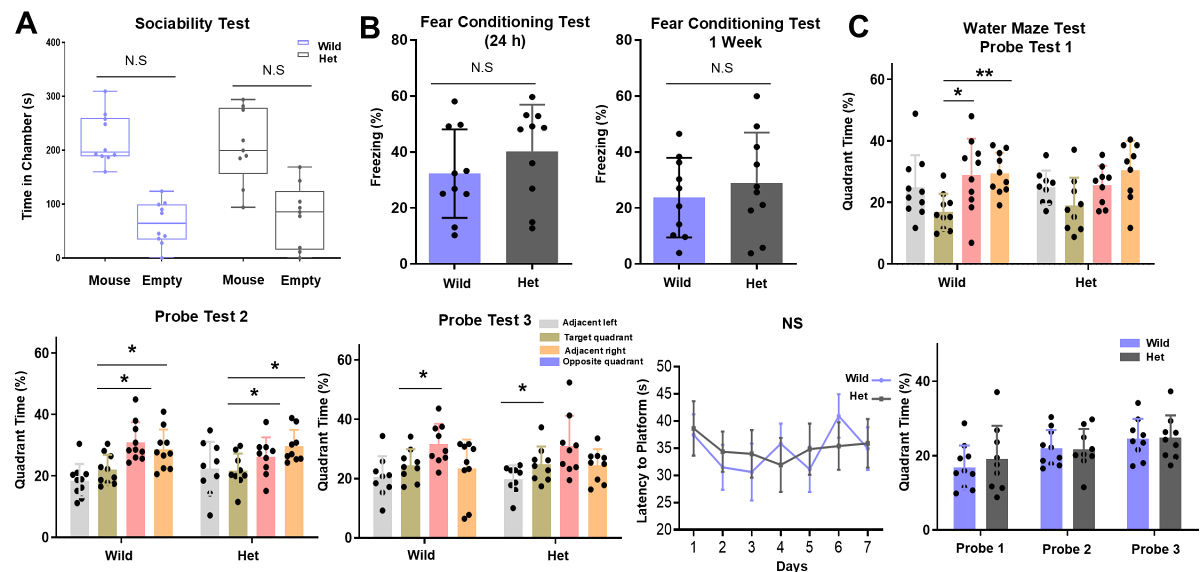

Supplement: Supplementary file 6 — Supplementary Material 6 [file 13041_2025_1238_MOESM6_ESM.tif]
